# Non-Coding RNAs as Mediators of Epigenetic Changes in Malignancies

**DOI:** 10.3390/cancers12123657

**Published:** 2020-12-05

**Authors:** Subhasree Kumar, Edward A. Gonzalez, Pranela Rameshwar, Jean-Pierre Etchegaray

**Affiliations:** 1 Department of Biological Sciences, Rutgers University, Newark, NJ 07102, USA; sree.kumar@rutgers.edu (S.K.); eag170@scarletmail.rutgers.edu (E.A.G.); 2 Department of Medicine, Hematology/Oncology, New Jersey Medical School, Rutgers Biomedical and Health Sciences, Newark, NJ 07103, USA

**Keywords:** ncRNAs, miRNAs, lncRNAs, epigenetics, cancer

## Abstract

**Simple Summary:**

This review discusses the role of non-coding RNAs (ncRNAs) in cancer epigenetics, mostly focusing on how deregulated microRNAs (miRNAs) and long non-coding RNAs (lncRNAs) alter the expression of cancer-promoting genes by targeting epigenetic factors to facilitate cellular malignancy. The potential for using ncRNAs as targets for early prognosis and for developing cancer therapies to be used in conjunction with current treatments is discussed.

**Abstract:**

Non-coding RNAs (ncRNAs) are untranslated RNA molecules that regulate gene expressions. NcRNAs include small nuclear RNAs (snRNAs), small nucleolar RNAs (snoRNAs), ribosomal RNAs (rRNAs), transfer RNAs (tRNAs), circular RNAs (cRNAs) and piwi-interacting RNAs (piRNAs). This review focuses on two types of ncRNAs: microRNAs (miRNAs) or short interfering RNAs (siRNAs) and long non-coding RNAs (lncRNAs). We highlight the mechanisms by which miRNAs and lncRNAs impact the epigenome in the context of cancer. Both miRNAs and lncRNAs have the ability to interact with numerous epigenetic modifiers and transcription factors to influence gene expression. The aberrant expression of these ncRNAs is associated with the development and progression of tumors. The primary reason for their deregulated expression can be attributed to epigenetic alterations. Epigenetic alterations can cause the misregulation of ncRNAs. The experimental evidence indicated that most abnormally expressed ncRNAs impact cellular proliferation and apoptotic pathways, and such changes are cancer-dependent. In vitro and in vivo experiments show that, depending on the cancer type, either the upregulation or downregulation of ncRNAs can prevent the proliferation and progression of cancer. Therefore, a better understanding on how ncRNAs impact tumorigenesis could serve to develop new therapeutic treatments. Here, we review the involvement of ncRNAs in cancer epigenetics and highlight their use in clinical therapy.

## 1. Introduction

The pathophysiology of cancer is associated with multiple molecular and cellular dysfunctions, including genetic and/or epigenetic alterations [1]. Epigenetic changes are catalyzed by specific enzymes capable of modifying the chromatin structure, e.g., acetylation, methylation, phosphorylation, ubiquitylation and glycosylation [2,3]. These modifications can be influenced by environmental changes [4,5]. The cellular epigenetic landscape can be regulated by non-coding RNAs (ncRNAs) to modify gene expressions [6,7,8]. ncRNAs are untranslated RNA molecules capable of regulating gene expression through multiple pathways. For instance, ncRNAs can target the chromatin to induce gene silencing through direct interaction with epigenetic factors. ncRNAs can also interact with transcription factors to either prevent or promote the expression of target genes. Additionally, ncRNAs can silence gene expressions by directly binding to mRNA targets, a process known as RNA-induced silencing. This review article is focused on the epigenetic function of small and long ncRNAs: microRNAs (miRNAs), short interfering RNAs (siRNAs) and long non-coding RNAs (lncRNAs) in the context of cancer biology.

In general, miRNAs typically repress specific gene targets by RNA-induced silencing at the post-transcriptional level [9]. In contrast, lncRNAs can use multiple methods to regulate gene expressions. These include the remodeling of chromatin to activate or repress transcription, modulating pre-mRNA splicing and inhibiting mRNA translation [10]. In addition to miRNAs and lncRNAs, other ncRNAs have specialized functions. These include small nuclear RNAs (snRNAs), small nucleolar RNAs (snoRNAs), ribosomal RNAs (rRNAs), transfer RNAs (tRNAs) and piwi-interacting RNAs (piRNAs). snRNAs are approximately 150 nucleotide bases and are primarily located within the splicing regions of nuclei. Due to their locations, snRNAs have been reported to be associated with pre-mRNA splicing, particularly in the formation of the spliceosome [11]. The spliceosome is a large, dynamic complex that is composed of the five snRNAs: U1, U2, U4, U5 and U6, along with other protein components [11,12,13]. The combination of snRNAs and other splicing proteins, a small nuclear ribonucleoprotein or snRNP complex, includes a singular spliceosomal snRNA within a complex with various splicing proteins [11]. Similar to snRNAs are snoRNAs; there is documentation of a group of ncRNAs primarily located in the nucleoli of eukaryotic cells with functions to modify and contribute to the processing of rRNA, particularly during the synthesis of the ribosomal subunits [14,15,16,17]. The two main functions of snoRNA in relation to rRNA modifications involve methylation of the ribosomal subunits or 2′O-ribose-methylations and pseudouridylation to convert uridine into pseudouridine for the generation of mature rRNAs [18]. rRNAs and tRNAs are long and well-studied ncRNAs with known specific functions in mRNA translation. rRNAs are known to have a role in the assembly of ribosomal subunits and tRNAs in protein synthesis, particularly in the transfer of individual amino acid subunits into the ribosome during the translation of mRNA [19,20]. Notably, small RNA fragments derived from tRNAs can function as ncRNAs, as described below. The function of piRNAs is predominantly linked to transposons, particularly to protect the genome from invasive transposable elements in the germlines of animals through gene silencing [21]. This ncRNA is approximately 24–32 nucleotide bases and is transcribed from a series of repetitive elements within the genome known as piRNA clusters [22]. piRNAs interact with PIWI, a subfamily of ARGONAUTE proteins, to regulate their targets, including self-biogenesis [23]. In addition to the silencing of transposable elements, piRNAs also regulate DNA rearrangements, mRNA turnover and epigenetic programming [23,24]. Overall, both miRNAs and lncRNAs can function as key components of epigenetic modulations to alter gene transcription in response to intracellular and extracellular cues. The expression of ncRNAs is deregulated in a variety of cancer types promoting tumor growth, invasion and metastasis. Thus, ncRNAs are potential therapeutic targets for cancer treatment. Here, we discuss how miRNAs and lncRNAs regulate the epigenome as part of cancer pathophysiology. 

## 2. MiRNA-Mediated Epigenetic Mechanisms 

MiRNAs are a class of double-stranded RNAs (dsRNAs) that are approximately 22 nucleotide bases. This class of ncRNA is mostly responsible to silence mRNA translation by direct interaction with the transcript [25]. MiRNAs exert multiple cellular processes, including cell proliferation, adhesion, cell death and differentiation [26]. They were first characterized in *Caenorhabditis elegans*, with the discovery of *lin-*4, which was shown to silence *lin-*14 mRNA, which is a protein involved in the initial stage of larval (L1) development [27]. 

**Figure 1:**
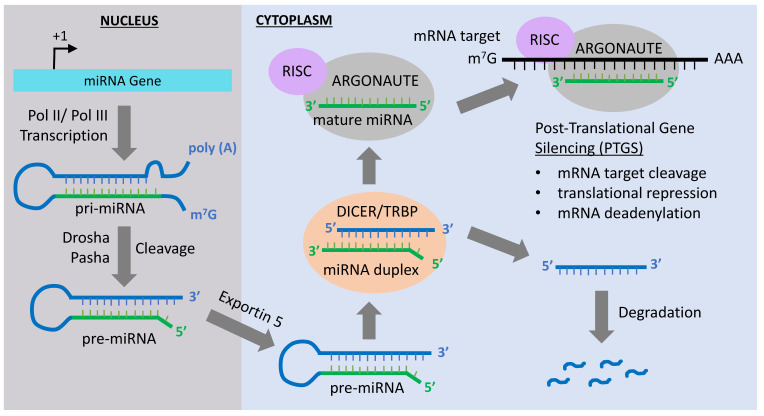
MicroRNA (MiRNA) biogenesis pathway. Transcription of the miRNA gene by RNA polymerase Pol II or Pol III produces a primary transcript (priRNA) that is cleaved by the ribonuclease III Drosha and processed by the double-stranded RNA-binding protein Pasha in the nucleus. This results in the formation of a precursor miRNA (pre-miRNA) hairpin, which is exported from the nucleus via exportin-5-mediated translocation. Once in the cytoplasm, the pre-miRNA is cleaved by the RNase DICER in a complex with the double-stranded RNA-binding protein TRBP to generate a miRNA duplex. The non-functional strand of the miRNA duplex is subjected to degradation, while the mature miRNA (functional strand) binds to ARGONAUTE proteins and the RNA-induced silencing complex (RISC). The mature miRNA guides RISC to silence mRNA targets by cleavage, translational repression or deadenylation.

MiRNA gene transcription forms a stem-loop, double-stranded structure known as primary miRNA (pri-miRNA) in the nucleus (Figure 1) [28]. The pri-mRNA is processed, in the nucleus, by the RNase III enzyme Drosha [29] and its cofactor, Pasha, resulting in the formation of pri-miRNA into pre-mRNA. The pre-mRNA is further processed by the ATP-dependent protein/enzyme Dicer into a mature miRNA [30,31,32,33]. Once in the cytoplasm, one of the two strands of the mature miRNA is taken up by a member of the ARGONAUTE protein family. ARGONAUTE then delivers the miRNA to a target mRNA sequence, causing its degradation or preventing its translation [34]. ARGONAUTE forms complexes with different proteins in order to deliver the miRNA to its targets. For instance, the heat-shock protein 90 (Hsp90) can form a complex with the ARGONAUTE 1 (AGO1) in plants, along with the single miRNA strand [35]. This overall complex is known as the RNA-induced silencing complex or RISC. The single miRNA strand either binds with perfect or imperfect complementarity to the mRNA target, generally in the 3′ UTR, which leads to translation repression or mRNA degradation [36]. 

SiRNAs are also small RNAs, commonly used experimentally as RNA interference or RNAi to silence genes. The use of RNAi was originally implemented in *C. elegans* in order to decrease the expression of specific genes and, since then, has been widely applied in varied experimental conditions [37]. This study found that double-stranded RNA interference (dsRNAi) molecules could be relatively more effective in silencing genes as compared to single-stranded siRNA. Shortly after this pioneering finding by Fire and colleagues in 1998, RNAi was implemented in the form of short hairpin RNAs (shRNAs) produced in plasmid vectors [38,39]. This followed the wide use of shRNAs through cell engineering with genomic-integrated shRNA [40]. Both siRNAs and shRNAs are used to silence genes for in vitro and in vivo experiments. In contrast to siRNAs, shRNA constructs are capable of DNA integration. After transcription, shRNAs are exported from the nucleus and recognized by Dicer in the cytosol to be processed into siRNA duplexes [41].

Post-transcriptional gene silencing (PTGS) and mRNA degradation allow miRNAs to regulate the epigenome through the downregulation of key epigenetic modifiers and to change the chromatin landscape [42]. Key examples of miRNA-interacting epigenetic factors include histone deacetylases (HDACs), histone methyltransferases (HMTs) and DNA methyltransferases (DNMTs) [42,43]. A list of miRNAs targeting specific repressive epigenetic modifiers is shown in Table 1. Thus, the upregulation of these miRNAs can specifically reactivate genes whose expressions were silenced by such epigenetic modifiers targeted by specific miRNAs. For instance, the miR-29 family has complementarities to the 3′ UTRs of DNMT3A and DNMT3B [44], resulting in their repression at the PTGS level. Similarly, miR-148 targets DNMT3B not at the 3′ UTRs but at the conserved protein-coding region [45]. Consequently, miR-148 interactions with DNMT3B mRNA lead to both PTGS, which could lead to translation deficiency or mRNA degradation [45]. DNA-methyl transferase 1 (DNMT1) is targeted by multiple miRNAs, including miR-148a, miR-152, miR-185 and miR-342. Many of the miRNAs listed in Table 1 have been reported to interact and repress the expression of the HMT, EZH2 (Enhancer of zeste homolog 2), which is part of the polycomb group repressive complex 2 (PRC2) [46]. EZH2 is targeted by several miRNAs, including miR-101, miR-137, miR-26a, miR-98, miR-124, miR-214 and let-7. In cases when miRNAs capable of suppressing DNMT1, DNMT3A, DNMT3B and EZH2 are decreased, it could lead to abnormal DNA methylation patterns to silence specific gene targets, resulting in cancer [47]. Abnormal DNA/histone methylation patterns could lead to the reactivation of oncogenes and/or the repression of tumor suppressors to facilitate cancer formation, progression and metastasis.

In addition to miRNAs capable of regulating the epigenome, the expression of such miRNAs themselves can be regulated by epigenetic modifications. For instance, CpG islands, which are generally found at gene promoters, are also found in approximately half of all miRNA genes, which can consequently undergo aberrant DNA methylation and deregulated expression [42]. These modifications can lead to either the upregulation or downregulation of miRNA expressions that can be associated with different states of tumorigenesis.

## 3. LncRNA-Mediated Epigenetic Mechanisms

LncRNAs are approximately 200 nucleotide bases in length and can be produced during the transcription of noncoding and protein-coding genomic regions. LncRNAs are mostly known in the context of gene repression. However, additional lncRNA roles include organizing the 3D genome, sequestering proteins for regulating gene expressions at the level of transcription or serving as scaffolding for the recruitment of proteins to specific genomic loci [51,52,53,54,55]. Thus, lncRNAs can be distinguished based on their molecular functions. For instance, lncRNAs can function as: (1) signals through the activation or repression of genes, (2) guides to bring chromatin modifiers to specific genomic loci, (3) decoys by displacing transcriptional repressors, (4) scaffolds for multiple protein complexes [56] and (5) competing endogenous RNAs (ceRNAs) [57]. Interactions between lncRNAs and epigenetic modifiers are highly relevant in the context of cancer, known for a vast number of epigenetic aberrations. 

Physiologically relevant roles for lncRNAs include X-chromosome inactivation, imprinting and the general remodeling of the chromatin landscape [58]. X-chromosome inactivation is controlled by the lncRNA XIST, originally studied in mice and humans. XIST is approximately 15–17 kb in length and exclusively located in the nucleus [59,60]. XIST has multiple regions that serve as protein-binding domains, which confers them the ability of binding multiple factors to regulate gene expressions. The process of X-chromosome inactivation occurs in females as a mechanism of gene dosage compensation to equalize the gene expression with males having one X-chromosome. The XIST-mediated inactivation of the X-chromosome can occur in two ways—random X-inactivation or imprinted-mediated X-inactivation [61]. XIST is able to inactivate X-chromosome genes due to its ability to recruit repressive epigenetic factors such as HDACs, PRC1 and PRC2-involved chromatin compaction, which results in gene repression [62,63]. Lamin B receptor (LBR), a critical protein required for tethering chromatin to the nuclear lamina, is important for the localization of XIST [64]. In addition to XIST, various lncRNAs interact with epigenetic modifiers to remodel the chromatin and regulate the gene expression. The Homeobox transcript antisense RNA (HOTAIR) is a well-studied lncRNA involved in regulating the epigenome whose expression is dysregulated in multiple cancers. It can bind to histone modifiers, such as the REST/CoREST/LSD1 complex and PRC2, which modify histone methylation at gene promoters. More specifically, the binding between HOTAIR and the REST/CoREST/LSD1 complex results in the methylation of histone H3 at lysine 4 (H3K4me), while the HOTAIR-PRC2 complex results in the methylation of histone H3 at lysine 27 (H3K27me), which are associated with the activation and silencing of genes, respectively [65]. The aberrant expression of HOTAIR impairs the functionality of such epigenetic modifiers. Upregulated HOTAIR results in the hypermethylation of H3K4 and/or H3K27, resulting in key gene targets to be activated and/or silenced, respectively. On the other hand, the downregulation of HOTAIR causes the hypomethylation of H3K4 and/or H3K27, leading to the deregulation and/or de-repression of its target genes. The accumulation of these epigenetic abnormalities leads to cancerous phenotypes. Unlike the ability of HOTAIR to work in conjunction with its corresponding epigenetic factors, other lncRNAs, such as SCHLAP1 (second chromosome locus associated with prostate-1), can directly affect the recruitment of the ATP-dependent chromatin-remodeling complex SWItch/Sucrose Non-Fermentable (SWI/SNF). SCHLAP1 inhibits the genomic recruitment of SWI/SNIF [66,67]. The SWI/SNF complex facilitates the recruitment of transcription factors by opening the chromatin [68]. Thus, mutations and/or the irregular expression of SCHLAP1 can affect the binding of SWI/SNF to chromatin and, consequently, disable the recruitment of transcription factors, resulting in deregulated gene expression. Along with SCHLAP1, the lncRNAs UCA1 (urothelial carcinoma associated 1) and NEAT1 (nuclear paraspeckle assembly transcript 1) can also alter the recruitment of SWI/SNF by binding to BRG1, a SWI/SNF subunit capable of activating transcription [69,70]. Another lncRNA, ANRIL (anti-sense non-coding RNA in the INK locus), can interact with PRC1 and PRC2 complexes to repress gene transcription, including tumor-suppressor genes such as the p15/CDKN2B, p16/CDKN2B and p14ARF gene clusters [71,72]. In addition to being repressed by miRNAs, EZH2, the catalytic subunit of PRC2, can interact with several lncRNAs, including HOTAIR, GAS5, MEG3, MALAT1 and KCNQ1OT1 [73]. These lncRNAs facilitate the recruitment of EZH2 at gene promoters to repress transcription [74]. The lncRNA PVT1 (plasmacytoma variant translocation 1) can also interact with EZH2, influencing H3K27me patterns of key genes, such as the angiopoietin-like 4 (ANGPTL4) [75]. Related to methylation, the lncRNA H19 can interact with S-adenosylhomocysteine hydrolase (SAHH), an enzyme required for the regeneration cycle of S-adenosylmethionine (SAM), which is the major donor of methyl groups during methylation [76]. Thus, alterations in the level of H19 can change global methylation patterns. 

The deacetylation of histones, which might preclude repressive histone methylation marks, leads to compaction of the chromatin landscape, resulting in gene repression. The lncRNA MALAT1 (metastasis-associated lung adenocarcinoma transcript 1) can form a complex with HDAC9 and the chromatin remodeling enzyme BRG1, which results in the dysfunction of smooth muscle tissue and contributes to thoracic aortic aneurysms [77]. This highlights the widespread involvement of lncRNAs and their interactions with key epigenetic modifiers in regulating the expression of multiple target genes. Table 2 summarizes the discussed lncRNAs and their interactions with epigenetic modifiers. This relationship is a critical aspect in cancer epigenetic mechanisms, since variations in lncRNA levels can alter the expression of key epigenetic genes associated with multiple states during carcinogenesis. 

## 4. MiRNAs in Solid Tumors

A growing amount of evidence supports the role of miRNAs in cancers. Within the last two decades, it has been shown that either the upregulation or downregulation of miRNAs correlate with the progression of both hematological and solid tumors (Table 3). Shown are the upregulated or downregulated miRNAs in multiple solid tumors.

Solid tumors can be metastatic, and such processes, including malignant cell survival, can be partly due to deregulated miRNAs (Table 3). For instance, miR-15b and miR-16 are upregulated in gastric cancer and correlate with a poor prognosis [79]. The expressions of miR-15b and miR-16 are linked to the upregulation of the antiapoptotic protein BCL2, resulting in decreased apoptosis or programmed cell death in gastric cancer cells [79]. In contrast, both miR-15b and miR-16 were found to induce apoptosis by the direct targeting of BCL2 in a leukemia cell line, suggesting varied roles for miRNAs in specific cancers [80]. Another miRNA, LET-7, is downregulated in lung cancers, which correlates with a poor patient prognosis [81]. Concomitantly, the overexpression of LET-7 can reduce metastatic lesions [81]. In terms of its epigenetic involvement, it was shown that deficiency of the histone deacetylase SIRT6 increased the progression of pancreatic cancer and metastasis due to upregulation of the oncofetal protein Lin-28, which is a negative regulator of LET-7 miRNA [82].

Both miR-21 and miR-34, with opposing roles, are dysregulated in several solid tumors [83,85,86,87,90,91,92]. Upregulated miR-21 in several cancers (Table 3) can be classified as a proto-oncogene and can be used as a biomarker of malignancy. Additionally, miR-21 is involved in the maintenance of pluripotency and can promote epithelial-to-mesenchymal cellular transitions (EMTs), with both roles associated with the process of cancer initiation [90]. Additionally, high levels of miR-21 correlate with increased tumor cell proliferation and invasion in colon cancer [91] and in breast cancer proliferation and metastasis [92,125]. Conversely, the miR-34 family was found to be downregulated in multiple cancers and are therefore considered as tumor suppressors [83]. The miR-34 family of miRNAs is expressed predominantly in the lungs, brain and the gastrointestinal tract and was shown to be part of a positive feedback with the tumor suppressor p53 to induce cell cycle arrest and apoptosis, thereby inhibiting tumorigenesis [85,86,87]. Targets of miR-34 include BCL2, CDK4/6 and cyclin E2, which are involved in blocking apoptosis or promoting cell cycle progression, thereby facilitating tumorigenesis [84,86]. Additionally, low levels of miR34a correlates with breast cancer aggressiveness and decreased patient survival. Mechanistically, miR-34a targets the stem cell-associated transcription factors E2F1/E2F3, which are upregulated in breast cancer patients [126]. 

MiRNAs are also known to repress key epigenetic regulators, and as discussed above, the outcomes of miRNA deregulation are associated with malignancies. These miRNAs (Table 1) are either upregulated or downregulated in cancer, causing epigenetic irregularities (Table 3). For instance, the miR-29 family is downregulated in lung cancer and is therefore considered a tumor suppressor due to its ability to inhibit DNA methylation, which causes the reactivation of tumor-suppressor genes [111]. Both in vitro and in vivo studies show that the forced expression of miR-29a can decrease the proliferation of lung cancer cells lines by repressing the expression of the DNA methyltransferases DNMT3A and DNMT3B [44]. 

The two most common cancers that are affected by deregulated miRNAs are colorectal cancer and glioblastoma multiforme (GBM). Additionally known as bowel or colon cancer, colorectal cancer has become a leading cause of death predominantly in Western countries, with a 4–5% probability of malignancy [127]. A large number of miRNAs are associated with the formation and progression of colorectal cancer, including miR-21, miR-30a, miR-34a and miR-145. As mentioned above, miR-21 is involved in various cancers. In colorectal cancer, miR-21 downregulates the tumor suppressor PDC4 at the post-transcriptional level, thereby stimulating cancer invasion and metastasis [91]. MiR-30a-5p is downregulated in colorectal cancer and can suppress tumor metastasis by targeting integrin ß3 (ITGB3) [110], which is overexpressed in colorectal cancer [128,129]. Integrins are transmembrane receptors involved in cell-to-cell adhesions and mediate critical signal transduction pathways such as mitogen-activated protein kinase (MAPK) to control cell proliferation, migration and survival [110,128,129]. A recent study showed that the upregulation of miR-34a was linked to increased survival in colorectal cancer patients [88]. Interestingly, the upregulation of miR-34a in colorectal cancer correlates with a decreased expression of *Period1* (PER1) and *Period 2* (PER2) genes, which are core components of the circadian clockwork mechanism [88,89,130]. MiR-145 is also downregulated in colorectal cancer [112]. A recent study confirmed that miR-145 is involved in the inhibition of cancer cell migration and invasion through the p21-activated kinase 4 (PAK4)-dependent pathway [113]. PAK4 is an essential kinase involved in cytoskeletal reorganization, which is an important step for cell migration [113]. MiR-145 specifically suppresses the migration and subsequent invasion of colorectal cancer cells by the direct inhibition of PAK4 [113]. 

GMB is the most aggressive brain cancer, with very limited treatment options. Surgery is the most commonly used method to remove GBM tumors but with poor prognosis [131]. Radiotherapy and chemotherapy with temozolomide are the most common/frontline nonsurgical treatments [132]. Several miRNAs are involved in the progression of GBM and resistance to chemotherapy. Thus, a better understanding of their mechanisms could facilitate the development of new treatment options. Some miRNAs involved in GBM include miR-7, miR-221, miR-125b, miR-181d, miR-648, miR-185 and miR9. MiR-7 is downregulated in GBM and is known to play a role in the inhibition of glucose metabolism and cell growth through regulation of the IGF-1R/Akt signaling pathway, which is essential for cellular proliferation [119]. In contrast, miR-221 is upregulated in GBM, causing the increased proliferation of glioma cells by targeting BIRC1, a neuronal inhibitor of apoptosis [109]. The upregulation of miR-125b increases cellular proliferation and inhibits apoptosis by directly targeting the p53 and p38 MAPK pathways, thereby functioning as an oncogene in GBM [97]. In vitro studies showed that the knockdown of endogenous miR-125b increases apoptosis and decreases cell proliferation in GBM [97]. miR-181d, which is downregulated in gliomas, targets *O*^6^-methylguanine DNA methyltransferase (MGMT), a protein that is critical for maintaining genomic stability [100,101,133,134,135]. MGMT can be epigenetically silenced upon methylation of its promoter, which is associated with the longer survival of GBM patients treated with temozolomide, a drug that methylates DNA at *O*^6^-methylguanine, causing DNA damage and the death of tumor cells [136,137,138]. Similar to miR-181d, miR648 was thought to affect the expression of MGMT [102]. However, later studies showed that transfected glioma cell lines with miR-648 did not show a suppression of MGMT expression [139] but, instead, a negative correlation between the MGMT expression and miR-648 [93]. As mentioned previously, miR-185 targets DNMT1 and, in doing so, regulates global DNA methylation. This is prevalent in GBM, because perturbations in DNA methylation are commonly associated with cancerous phenotypes [140,141]. A study that looked at the relationship between methylation and GBM found multiple hypermethylated genomic regions that were affected by the downregulation of miR-185 [120]. The same study found DNA hypermethylation at gene promoters in primary glioma cell lines [120]. Some of these hypermethylated genes included histone H3.1 (HISTH3E), glutamate decarboxylase (GAD1) and ankyrin repeat and death domain-containing protein 1A (ANKDD1A). Hypermethylation, as previously mentioned, correlates with a closed chromatin configuration and is therefore linked to gene repression. Collectively, some miRNAs target single proteins affecting key cellular pathways, while other miRNAs target epigenetic regulators, which have greater impact due to their ability to alter the expressions of multiple genes, as seen with miR-185. Another miRNA involved in brain tumorigenesis is miR9. This miRNA is upregulated in GBM patients and causes a resistance to temozolomide, a drug currently used for GBM treatment that targets the Sonic Hedgehog receptor PTCH1 [142]. 

## 5. MiRNAs in Hematologic Malignancies

Hematologic malignancies are those that derive from hematopoietic cells, also referred to as blood-related cancers. Such malignancies include chronic lymphoid leukemia (CLL), multiple myeloma, acute lymphoblastic leukemia (ALL), acute myeloid leukemia (AML), Hodgkin’s lymphoma, cutaneous T-cell lymphoma (CTCL) and non-Hodgkin’s lymphoma (NHL).

The role of miRNAs in healthy and malignant hematopoietic processes has been extensively studied, and it is this fact that links miRNAs to the formation of hematological malignancies. For instance, miRNAs involved in hematopoietic differentiation through a translational control of targeted mRNAs include miR-128a and miR-181a [95,96,103,143,144,145,146]. These miRNAs control the differentiation of multipotent progenitor cells (MPPs) to form distinct lineages with the myeloid and lymphoid pathways. MiR-128a targets Lin28, which is highly expressed in embryonic stem cells, and has been shown to sustain cancer stem cells [143,144,145]. MiR-125b, another miRNA targeting Lin28, is involved in the gradual development of myeloid leukemia [95,96]. However, the role of miRNAs is complex, with multiple miRNAs targeting a distinct stage of hematopoietic development. 

Upregulated miR-181a increases the proliferation of the B-lymphoid cells, while miR-17, miR-24a and miR-155 are linked to early stages of myeloid differentiation [103,146]. Mechanistically, miR-17 inhibits p21 and STAT3, which are key players in cell cycle arrest, and can induce the differentiation of myeloid cells through the HIF-1α (hypoxia-inducible transcription factor 1α)-mediated differentiation of AML cells [118]. 

The ectopic expression of miR-24 promotes the survival of both myelopoiesis and lymphopoiesis by blunting apoptosis by targeting proapoptotic proteins, including caspase 9 (Casp 9) [147,148,149]. An increased expression of miR-24 is observed in AML and Hodgkin’s lymphoma (HL) [150,151]. Interestingly, the enforced expression of miR-155 exhibits dual roles—oncogenic and tumor-suppressive in AML [104]. Overall, miRNAs involved in hematopoiesis can impact cell proliferation, differentiation and apoptosis, and thus, their dysregulation can contribute to hematological cancers. 

Additional key miRNAs involved in hematological malignancies include miR-125b, miR-150, miR-155 and the miR-17/92 cluster. MiR-125b is upregulated in numerous neoplastic hematological disorders, particularly in AML [152,153]. Mechanistically, miR-125b targets numerous downstream effectors involved in the cell cycle, differentiation and apoptosis [154]. One of these downstream effectors is the CCAAT/enhancer-binding protein-α (C/EBPα), which is frequently mutated in AML patients [155]. C/EBPα is a transcription factor and a tumor suppressor previously shown to prevent the expansion of myeloid progenitors [156]. Paradoxically, the upregulation of miR-125b can potentially be attributed to C/EBPα activity, as one recent study shows that miR-125b is a direct target of C/EBPα in AML [98]. Like many miRNAs, miR-125b affects the expression of multiple targets. For example, miR-125b can promote the expression of the vascular endothelial growth factor (VEGFA), which promotes angiogenesis and metastasis. The overexpression of miR-125b is able to increase the expression of VEGFA, in part by reducing the expression of ten-eleven translocation enzyme 2 (TET2), which is a critical epigenetic enzyme involved in an active DNA demethylation process mediated by successive DNA oxidations [99,157]. Liu and colleagues [99] also provided in vivo evidence, using an AML mouse model, that the overexpression of miR-125b can cooperate with MLL-AF9 (mixed-lineage leukemia (MLL) fused to the ALL1-fused gene from chromosome 9 (AF9)), a fusion protein associated with AML. MLL is a histone methyltransferase targeting H3K4, a modification associated with gene activation, and AF9 is part of the Super Elongation Complex (SEC) and one of the most common fusion partners of MLL [158,159,160]. Previous studies have also shown that miR-125b silences proapoptotic proteins, including Puma, Bak1 and Bmf, as well as p53, to induce cell cycle arrest [153]. In contrast to the upregulation of miR-125b, the downregulation of miR-150 was also correlated with the development and progression of hematological malignancies. For example, miR-150 is downregulated in AML, causing an increase in c-Myb and Flt3, which are key antiapoptotic proteins that promote cell proliferation [122]. Additionally, miR-150, which inhibits tumor invasion and metastasis, is downregulated in cutaneous T-cell lymphoma (CTCL) [123,161]. This study by Ito and colleagues provides in vivo evidence that miR-150 is capable of suppressing tumor metastasis and invasion by targeting the chemokine receptor or CCR6, which prevents autocrine signaling in advanced CTCL, particularly IL-22-CCL20-CCR6 signaling [123]. Another miRNA, miR-155, is overexpressed in multiple hematological malignancies and associated with poor survival in AML patients [105,106,107]. miR-155 is formed by RNA processing of the B-cell integration cluster (BIC), which is a non-coding gene upregulated in Hodgkin’s lymphoma [107,108]. miR-155 is also upregulated in AML, particularly in the subtype FLT3-ITD tumors, which is generally correlated with poor prognosis [105].

Interestingly, there is a polycistronic group of miRNAs, known as the miR-17/92 cluster within chromosome 13 in humans [162]. The miR-17/92 cluster, also referred to as “oncomiR-1”, is an oncogenic cluster of miRNAs that include miR-17, miR-18a, miR-19a, miR-20a, miR-19b-1 and miR-92a-1. This cluster is collectively involved in apoptosis, homeostasis and cell proliferation. The miR-17/92 cluster is regulated by various oncogenic transcription factors, including MYC, MYCN, STAT3 and E2F, and it represses key proteins involved in various cellular processes—for example: BCL3 (cell proliferation), E2F (G1/S cell cycle progression), CDKN1A (cell cycle arrest) and ZBTB4 (p53 response) [163]. miR-17/92 is also epigenetically regulated by the histone demethylase JARID1B, which targets di- and trimethylated histone H3 at lysine 4 (H3K4me2 and H3K4me3), thereby causing the silencing of this cluster [164,165,166]. The miR-17/92 cluster is often dysregulated in solid tumors and hematological malignancies [167,168]. Under normal conditions, in vivo studies in mice show the involvement of this cluster in hematopoiesis—for example: lymphocyte development (miR-17 and miR-19b-1) and B-cell differentiation and maturation [115,169]. miR-17 and miR-19b-1 are significantly upregulated in CLL [116] and exhibit a moderate upregulation of miR-18a, miR-19a and miR-92a [117]. Collectively, the role of these miRNAs is critical for regulating downstream effector proteins involved in cell migration, apoptosis, cell cycle arrest and proliferation, as well as epigenetic modulators. Thus, altering the levels of these miRNAs can disrupt the cellular physiology and homeostasis, resulting in cancer.

## 6. LncRNAs in Solid Tumors

**Figure 2:**
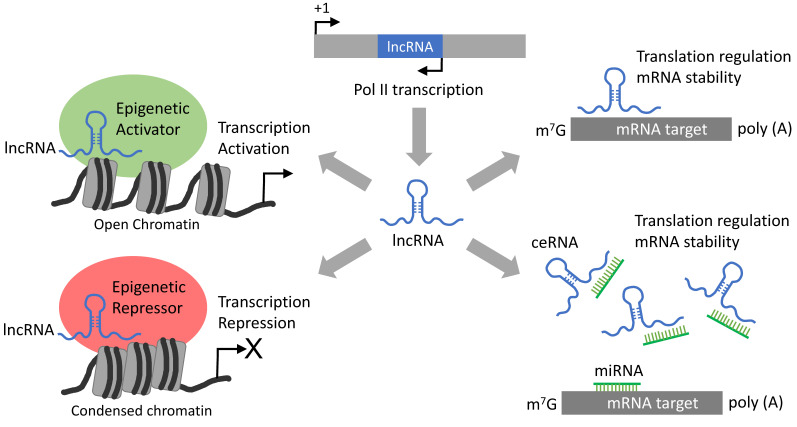
Long non-coding RNA (LncRNA)-mediated gene regulation mechanisms. Generally, lncRNAs originate from antisense transcripts produced by RNA polymerase II (Pol II). LncRNAs can be produced from diverse genomic locations, including introns, coding regions and sequences between genes. LncRNAs can recognize specific mRNA targets to modulate their expression by affecting post-transcriptional processes, including translation and mRNA stability. Alternatively, lncRNA can recruit proteins such as epigenetic regulators (activators or repressors) as a scaffolding system guided to regulate the expression of specific target genes. Additionally, lncRNAs can regulate specific gene expressions by functioning as competing endogenous RNAs (ceRNAs) capable of sequestering miRNAs.

LncRNAs are transcribed by Pol II from either coding or non-coding sequences, resulting in over 200 nucleotide RNA sequences forming specific three-dimensional conformations, which enables them to interact with specific proteins, such as epigenetic regulators to modulate gene expressions (Figure 2). Alternatively, lncRNAs can regulate translation and mRNA stability by direct binding to target mRNAs or by functioning as competing endogenous RNAs (ceRNAs) to quench specific miRNAs (Figure 2). 

Similar to miRNAs, the expression of lncRNAs is affected in multiple types of cancers. The altered expressions and/or mutations of lncRNAs facilitate the formation of tumors and, subsequently, lead to metastasis [170]. Either the upregulation or downregulation of lncRNAs can have negative effects on key downstream targets, including epigenetic regulators, thereby altering the expression of numerous genes. LncRNAs are involved in multiple oncogenic pathways, and their dysregulation affects cellular survival. Table 4 lists key examples of lncRNAs involved in the formation of solid tumors. Note that some of the lncRNAs in this list overlap with those listed in Table 2, further highlighting the fact that the dysregulation of lncRNAs can be a consequence of epigenetic alterations. 

PVT1 is a lncRNA amplified in human cancers, particularly in gastrointestinal tumors, and associated with poor prognosis [171]. PVT1 has several different isoforms exhibiting differential expression patterns, which are linked to multiple cellular pathways [171]. For instance, *PVT1* functions as a competing endogenous RNA (ceRNA) for miRNAs [209,210]. Thus, PVT1 can quench the function of several miRNAs, inhibiting their activity and thereby affecting the invasive and proliferative capacity of tumor cells [172]. Some of these miRNAs include miR-30a, miR-186 and miR-128 [211,212,213]. The downregulation of these miRNAs can promote tumorigenesis and metastasis. Furthermore, the location of the PVT1 gene is considered to be a cancer risk genomic locus, primarily because it shares such a region with MYC, which is a well-studied oncogene [214]. Moreover, the stability and activity of MYC is potentiated when partnered with PVT1 [215]. In addition to PVT1, the lncRNAs HOXD1-AS1, HOTAIR and MALAT1 are also associated with multiple types of solid tumors. HOXD cluster antisense RNA 1 (HOXD1-AS1) is dysregulated in various tumor types (Table 4). The dysregulation of HOXD1-AS1 in a vast number of cancers increases the growth, migration and invasion of tumor cells [176,177]. Mechanistically, HOXD1-AS1 can suppress the growth of colorectal carcinomas and metastasis by inhibiting MAPK/AKT signaling (a crucial pathway involved in cell proliferation) and the HOXD3-mediated transcriptional activation of integrin ß3, which initiates MAPK/AKT signaling [178]. At the epigenetic level, HOXD1-AS1 represses the transcription of HOXD3 through the recruitment of PRC2, which induces the accumulation of the repressive epigenetic mark H3K27me3 on the HOXD3 promoter. In addition to HOXD1-AS1, HOTAIR is a well-studied lncRNA that is upregulated in a variety of cancer types (Table 4). HOTAIR is an intergenic lncRNA that functions as an oncogene by promoting tumor cell growth, invasion, metastasis and drug resistance [184]. Mechanistically, HOTAIR increases tumor cell proliferation in lung adenocarcinomas by reducing the expression of p21, an inhibitor of cell cycle progression at the G1 phase [179]. HOTAIR was proposed to function as a scaffold to facilitate the recruitment of PRC2 and LSD1/CoREST/REST epigenetic complexes to increase H3K27me3 and H3K4me2, respectively, on targeted genomic loci [216,217,218]. Since p21 expression is regulated by PRC2 in lung cancer cells, it is possible that the upregulation of HOTAIR can promote the recruitment of PRC2 to downregulate p21 expression in lung adenocarcinoma cells [219,220]. However, the artificial chromatin tethering of HOTAIR in breast cancer cells caused transcription repression independently of PRC2 [221].

HOTAIR is also upregulated in gastric cancer promoting tumor growth and metastasis and is therefore proposed as biomarker for poor prognosis in gastric cancer patients [180]. Notably, HOTAIR can also function as a ceRNA to derepress the expression of HER2 (human epithelial growth factor receptor 2), an oncogene that produces a growth factor receptor involved in augmenting cell proliferation and tumorigenesis [180,222]. Mechanistically, HOTAIR blocks the miR-331-3p-mediated downregulation of HER2 in gastric cancer [180]. miR-331-3p inhibits colorectal cancer cell growth by targeting HER2 [223]. Concordantly, the inhibition of HOTAIR reduces the progression and invasiveness of gastric cancer cells [224]. HOTAIR is also upregulated in cervical cancer, and its expression is activated by the transcription factor STAT3 [183]. As shown in Table 2, HOTAIR interacts with key epigenetic factors; therefore, its upregulation can result in the regulation of many downstream gene targets [182]. 

Another lncRNA whose expression is upregulated in multiple cancers is MALAT1 (metastasis-associated lung adenocarcinoma transcript 1). The knockdown of MALAT1 inhibits cervical cancer cell invasion, which occurs by blocking EMT in both in vitro and in vivo model systems [200]. The upregulation of MALAT1 promotes cancer cell growth and invasion, disables apoptotic pathways and correlates with a poor prognosis in cervical cancer patients [201]. Notably, MALAT1 can regulate mRNA splicing, transcription, ceRNA function and can interact with PRC2 to promote the methylation of H3K27 [202,225,226,227,228]. The upregulation of MALAT1 in a variety of cancers, which combines with its pleiotropic roles in gene regulation, has become the focus for therapeutic interventions of cancers [228]. 

Another lncRNA whose function is linked to epigenetic alterations is SChLAP1, capable of interacting with the ATP-dependent chromatin remodeling complex SWI/SNF (Table 2). The interaction with SChLAP1 antagonizes the tumor-suppressive function of SWI/SNIF, thereby promoting prostate cancer cell invasion and metastasis [66,229]. SNHG1 is a lncRNA whose expression correlates with aggressive cervical cancer by promoting tumor cell proliferation, migration and invasion [195]. Consequently, SNHG1 depletion reduced metastatic lesions in cervical cancer [195]. Overall, an upregulation of the lncRNAs HOTAIR, MALAT1 and SChLAP1 promote cancer cell proliferation and metastasis by interacting with transcription factors or epigenetic regulatory complexes to modulate gene expressions, which, in turn, facilitate the invasiveness and metastatic phenotypes of aggressive solid tumors.

LncRNA are also deregulated in other solid tumor types, such as breast and gastric cancers that exhibit deregulated levels of the lncRNAs [173]. SPRY4-IT1, GAS5, PANDAR and H19 are lncRNAs dysregulated in breast cancer. Upregulated SPRY4-IT1 (SPRY4 intronic transcript 1) promotes the proliferation of human breast cancer cells by increasing the expression of the transcription corepressor ZNF703 (zinc finger 703), which may function as an oncogene in breast carcinoma cells [186]. Related to cell proliferation, GAS5 dysregulation is involved in multiple signaling pathways, all of which correlate with the progression of breast cancer. For instance, GAS5 is downstream of the NOTCH signaling pathway, which can promote the proliferation of breast cancer cells [187]. Additionally, GAS5 is downstream of NODAL signaling, which is involved in cancer stem cells (CSCs) self-renewal [188,189]. PANDAR, is upregulation in breast cancer, and its silencing in vitro suppresses the transition of breast cancer cells from G1 to the S phase, resulting in a decrease in tumor cell proliferation [190]. Mechanistically, PANDAR suppresses the expression of the cell cycle regulator p16(INK4A) by recruiting the repressive epigenetic factor BMI1—a component of the polycomb repressive complex 1 (PRC1)-to-p16(INK4A) promoter (Table 2) [190]. H19 regulates the tumorigenesis and progression of breast cancer by modulating the gene expressions at multiple levels: transcriptional, post-transcriptional and epigenetically [197]. 

Gastric cancers show the deregulation of several lncRNAs, including MEG3, CCAT-1 and ANRIL. The downregulation of MEG3 results in decreased apoptosis and increase proliferation [194]. Possibly, MEG3 can suppress gastric cancer growth by inhibiting the EMT [194] or by reducing the stemness of gastric cancer cells, which has been reported to mediate gastric cancer progression [230]. The lncRNA CCAT-1 (colon cancer-associated transcript 1) contributes to the growth and invasion of gastric cancer cells by functioning as a ceRNA targeting miR-219-1 [196]. ANRIL is upregulated in tumorigenesis and prevents the miR-99a-mediated inhibition of BMI1 [205]. Additionally, ANRIL can directly interact with the PRC components (Table 2). Collectively, the misregulation of various lncRNA affect the diverse epigenetic pathways that, consequently, change gene expression programs, favoring tumor cell proliferation and invasion. 

## 7. LncRNAs in Hematologic Malignancies

The role of lncRNAs in hematological malignancies is closely linked with the role of lncRNAs in key hematopoietic processes. Some examples of known lncRNAs that are known to be involved in malignant hematopoiesis are GAS5, FAS-AS1 and LUNAR1. GAS5 regulates T-cell proliferation by suppressing DNA binding of the glucocorticoid receptor [231]. The FAS-AS1 lncRNA represses the expression of the FAS receptor, which promotes apoptosis [232]. The LUNAR1 (leukemia-induced non-coding activator RNA) lncRNA functions as an oncogenic RNA promoting T-cell acute lymphoblastic leukemia (T-ALL) by increasing the IGF1R mRNA levels to maintain IGF1 signaling and to increase cell proliferation [207]. 

Altered levels of the discussed lncRNAs can alter the hematopoietic homeostatic balance, leading to hematological malignancies. Another target of IGF1R is the lncRNA IRAIN, which is downregulated in AML [208]. The abnormal regulation of IGF1R is associated with the progression and therapeutic resistance of hematological cancers. IGF1R is one of the most phosphorylated receptors in AML, and this phosphorylation causes continuous activation of the PI3K/Akt signaling pathway, which promotes cellular growth. Located within the IGF1R gene locus, the IRAIN gene transcribes the IRAIN lncRNA that has the ability to interact with chromatin to form intrachromosomal enhancer/promoter loops [208]. Therefore, due to its genomic proximity to the IGF1R gene, the abnormal expression of IRAIN can interfere with the IGF1R signaling pathway. 

**Figure 3:**
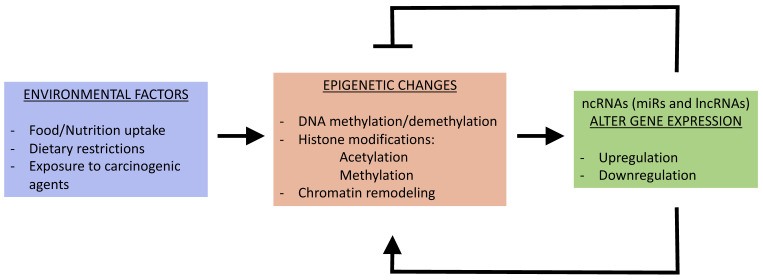
Non-coding RNAs (NcRNAs) in cancer. A decrease in cell cycle arrest and apoptosis, along with an increase in cell proliferation, are hallmarks of cancer cells associated with solid tumors and hematological malignancies. NcRNAs can regulate these cancer hallmarks by the direct targeting of cancer-promoting genes involved in the cell cycle, apoptosis and proliferation or by targeting epigenetic factors that modulate the expression of such genes. Additionally, lncRNAs can regulate the expression of cancer-promoting genes by functioning as scaffolds to recruit epigenetic factors or by acting as ceRNAs.

Deregulations of a wide range of lncRNAs have been implicated in hematological malignancies, as well as solid tumors. These include PVT1, HOTAIR, MEG3, UCA1, CCAT-1, ANRIL and NEAT1, while others such as LUNAR1 and IRAIN are only associated with blood cancers. In addition to solid tumors, PVT1 upregulation has been shown to be involved in acute promyelocytic leukemia and parallels MYC expression [174]. As described above, PVT1 and MYC are in adjacent genomic loci and could thereby influence each other’s expressions. The lncRNA HOTAIR can modulate the expression of c-KIT, a proto-oncogene, through competitive interaction with miR-193a [181]. MiR-193a is downregulated in AML, due to hypermethylation in the promoter region, and can increase cell proliferation [181]. Although the deregulation of HOTAIR is primarily associated with solid tumors, it is also upregulated in AML and correlates with a poor prognosis [233]. MEG3 is an imprinted gene, and its promoter hypermethylation can serve as biomarker for AML and MDS patients [192]. Hypermethylation and, therefore, the downregulation of MEG3 are associated with a poor prognosis [192]. Possibly, hypermethylation of the MEG3 promoter in AML is due to decreased TET2 activity [234]. The oncogenic lncRNA, urothelial carcinoma-associated 1 (UCA1), promotes tumor cell proliferation in AML [199]. The upregulation of UCA1 occurs upon inactivation of its transcription repressor C/EBPα (CCAAT/enhancer-binding protein α), which is mutated in AML, resulting in the expression of a dominant negative isoform (C/EBPα -p30) capable of inducing UCA1 expression [199]. The lncRNA CCAT1 (colon cancer-associated transcript 1) acts as a ceRNA to modulate the cell growth and differentiation in AML [235]. In AML patients, upregulated CCAT1 functions as a ceRNA targeting miR-155 to increase the MYC expression [235]. Notably, AML patients have decreased levels of miR-155 [235]. The lncRNA ANRIL was found to increase the proliferation of adult T-cell leukemia (ATL) cells by interacting with EZH2. ATL malignancy is caused by infection with human T-cell leukemia virus type 1 (HTLV-1) [236]. The binding of ANRIL to EZH2 (Table 2) results in activation of the nuclear factor kappa-light-chain-enhancer of activated B cells (NF-kB) pathway in ATL cells by promoting p65 binding to its target genes [206]. ANRIL is upregulated in HTLV-1-infected cells, leading to the increased proliferation of ATL cells [206]. LncRNA NEAT1 (nuclear enriched abundant transcript 1) is repressed in primary chronic myeloid leukemia (CML) cells [203]. The inhibition of NEAT1 impairs myeloid differentiation in acute promyelocytic leukemia (APL) cells, correlating with a malignant phenotype [203]. Collectively, either the upregulation or downregulation of lncRNAs can affect cell proliferation, cell cycle arrest and apoptosis, which, in turn, may favor carcinogenesis (Figure 3). In addition to assisting on epigenetic pathways, lncRNAs are subjected to epigenetic regulation 

## 8. Use of ncRNAs in Clinical Therapy

Alternative treatments for cancers are active areas of research aiming to combine or replace chemotherapy, immunotherapy and surgery. Gene therapy approaches involving CRISPR/Cas-9 and chimeric antigen receptor (CAR) T-cell therapy are promising for treating cancers [237,238,239]. RNAi can be used as a potential treatment for silencing overamplified, proto-oncogenic ncRNAs; however, using a siRNA-based technology comes with its challenges: a low cellular uptake, off-target effects, triggering of an immune response and a low efficiency in unstable physiological environments [240]. The premise is to deliver siRNAs with lipid-based nanoparticles or chemically modified siRNAs in combination with current anticancer drugs [240]. The advantages for implementing nanoparticles in RNAi delivery [241,242] include: (1) their small sizes, ranging from 10–1000 nm, (2) their ability to protect anticancer RNAi molecules from degradation, (3) to prevent an immune reaction and (4) higher transporting efficiency [242]. In addition to nanoparticles, lipid-based delivery mechanisms can offer promising advantages for RNAi delivery [243], including: (1) easy preparation, (2) the ability to load anticancer drugs on lipids in conjunction with RNAi molecules and (3) their physically stable structures [244]. A specific example of this delivery system in action is the use of lipid nanoparticles to deliver siRNA directly into the bloodstream of CML mice models [245]. The goal would be a delivery system that is efficient, minimally invasive and deprived of nonspecific target effects.

As highlighted in this review, alterations in the epigenetic modification of ncRNA genes can impact their expression. Thus, chemical inhibitors or activators targeting such epigenetic modifiers could serve as treatments against cancer. For instance, HDAC inhibitors could be used as a potential therapy against triple-negative breast cancer to derepress the expression of miR-200, which functions as a tumor suppressor by preventing EMT and metastasis [121,246]. MiR-200 is downregulated in breast cancer due to increased HDAC activity. Thus, a broad spectrum of HDAC inhibitors, such as sodium butyrate (NaB) and panobinostat (LBH589), could increase the expression miR-200 [121]. Another HDAC inhibitor, vorinostat, was shown to reduce metastasis in cutaneous T-cell lymphoma by targeting miR-150, which is downregulated in the early stages of this cancer [124]. Additionally, the use of DNMT inhibitors (DNMTi) for cancer therapy is a prevalent area of cancer research therapy [247]. However, the FDA-approved DNMTis decitabine and azacitidine show very limited reductions in solid tumors, despite showing a global increase in DNA demethylation [248,249]. Possibly, these DNMTis would be more effective in combination with other therapies. Another epigenetic target to be considered is EZH2 because of its association with numerous ncRNAs. Indeed, EZH2 inhibition showed therapeutic efficacy against ovarian, colon and bladder cancers [175,250]. Tazemetostat, an EZH2 inhibitor, showed encouraging results in a phase 1 clinical trial for treating relapsed or refractory B-cell non-Hodgkin’s lymphoma [251]. However, potential off-target effects and the inability to discern between normal and cancerous cells remain as major challenges for the use pharmacological agents to modulate the epigenome of tumor cells. 

One of the major challenges in cancer therapy is the acquisition of chemoresistance, which leads to cancer recurrence [252]. The dysregulated expression of miRNAs can promote cellular dedifferentiation into cancer stem cells (CSCs) capable of promoting therapeutic resistance and metastasis [253,254]. For example, miR-128, considered a tumor suppressor in lung cancer, inhibits CSC proliferation, thereby increasing the sensitivity to the chemotherapeutic drug paclitaxel [253]. Similarly, miR-181b, functioning as tumor suppressor in lung cancer, can decrease CSC self-renewal, causing chemosensitivity [253]. Thus, augmented CSC proliferation due to a low expression of miR-128 and/or miR-181b could result in chemoresistance, metastasis and lung cancer relapse. In contrast, other miRNAs such as miR-221 and miR-21 can promote CSC proliferation in colorectal and gastric cancers, respectively [253]. Thus, the upregulation of these miRNAs could promote metastasis and chemoresistance. Therefore, specific miRNA targeting in combination with current therapies could diminish the renewal of CSCs, thereby augmenting the chemosensitivity and prolonging the survival of cancer patients. Additionally, misregulated expressions of lncRNAs can confer chemoresistance. For instance, the upregulation of HOTAIR confers a resistance to tamoxifen, a conventional therapeutic for breast cancer patients, and is therefore a potential target for reversing chemoresistance [252,255]. 

Notably, tRNA-derived small RNA fragments (tsRNAs) can function as ncRNAs and were shown to be deregulated in both solid tumors and hematological malignancies [256,257]. For instance, overexpression of the tsRNAs *ts-46* and *ts-47* inhibit the growth and survival of lung cancer cell lines [256]. More recently, *ts-43* and *ts-44* were shown to be downregulated in chronic lymphocytic leukemia (CLL) and proposed to function as tumor suppressors [258]. In contrast, other tRNA-derived short non-coding RNA fragments called tRFs (tRF-3 and tRF-5) were shown to be upregulated in CLL and suggested to have an oncogenic role [258]. Thus, these newly discovered ncRNAs derived from tRNAs, functioning as tumor suppressors or oncogenes, add a new layer of complexity to the gene regulatory programs involved in malignancies. TsRNA expression levels could be used as diagnostic tools before and during cancer therapy to anticipate the drug resistance and relapse. Importantly, tsRNAs, which are similar to piRNAs, can interact with PIWI proteins to regulate the gene expressions via epigenetic modifications and can thereby serve as potential targets for developing new cancer treatments [259,260]. It would be informative to determine if dysregulated tsRNAs are involved in CSC renewal, metastasis and chemoresistance. 

Overall, ncRNAs are plausible targets for developing new anticancer therapies that could be used in combination with chemotherapeutic drugs to prevent the expansion of CSCs, metastasis, chemoresistance and cancer relapse. A potential strategy for targeting ncRNAs could include antisense oligonucleotides delivered by nanoparticles, a concept referred to as cancer nanomedicine, which is based on nanotechnology [261]. 

## 9. Conclusions

Overall, the aberrant expressions of miRNAs and lncRNAs correlate with cancerous phenotypes and the poor prognosis of cancer patients. Thus, understanding the mechanisms by which ncRNAs are expressed could facilitate the development of cancer therapies. As previously discussed, one of the primary causes of the upregulation and downregulation of ncRNAs can be attributed to epigenetic changes resulting in their abnormal expressions in a variety of cancer types. NcRNAs can modify the epigenome by interacting with epigenetic regulators or modifying their expression, which ultimately leads to changes in the gene expression that could favor the amplification of oncogenic pathways. Epigenetic alterations can be accumulated in response to environmental changes capable of altering the expression of ncRNAs, resulting in cancerous phenotypes [262,263,264,265]. For instance, mutations in ncRNA affecting epigenetic programs might have been formed upon exposure to carcinogenic agents [266]. Additionally, pharmacologically based anticancer treatments could impact the expression of ncRNAs to facilitate cancer recurrence, a possibility that remains to be determined. 

The expression of multiple miRNAs can be altered by epigenetic mechanisms leading to the misregulation of target genes and causing cancer. For instance, the upregulation of miR-21, observed in several cancers (Table 3), can be attributed to a loss of DNA methylation in its promoter [267]. DNA hypomethylation increases the expression of miR-21 and miR-146b in papillary thyroid carcinoma [267]. The expression of other miRNAs, such as miR-338-5p and miR-421, were proposed to be epigenetically silenced by EZH2 in prostate cancer [268], although in a H3K27me3 analysis, remained undetermined. 

**Figure 4:**
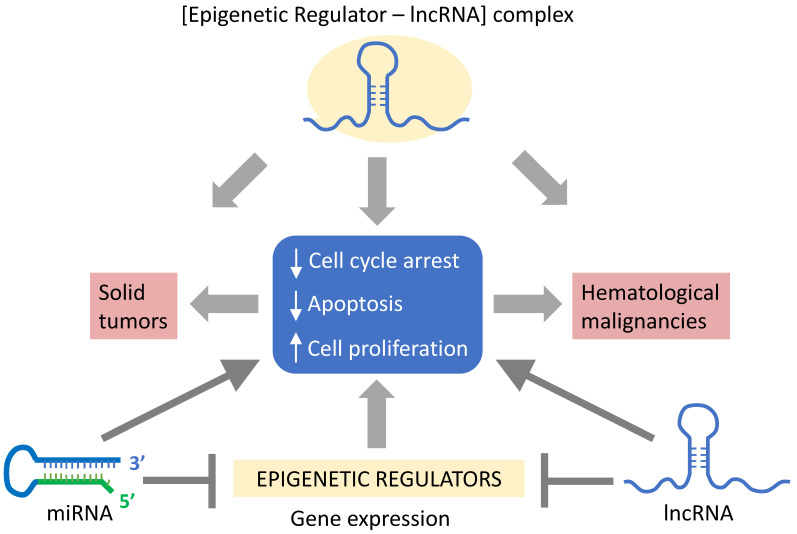
Environmental factors impact the link between the epigenome and ncRNAs. Environmental factors, including dietary changes and exposure to carcinogens, can affect the expression of epigenetic regulatory complexes targeting the regulation of ncRNAs. Epigenetic modulation can either upregulate or downregulate the expression of ncRNAs, which, in turn, feed back onto the epigenetic landscape, promoting the gene expression profiles involved in carcinogenesis.

Epigenetic mechanisms were also shown to control the expression of lncRNAs. For instance, the upregulation of the lncRNA NEAT1 in gastric cancer was attributed to a decrease in methylation by the RNA demethylase ALKBH5 (alkylation repair homolog protein 5) [204]. Furthermore, the binding of NEAT1 and ALKBH5 can negatively affect the expression of EZH2, thereby promoting tumor cell invasion and metastasis [204]. HOTAIR is deregulated in various cancers and can serve as a scaffolding to facilitate the recruitment of the PRC2 and LSD1/CoREST/REST complexes [218] (Table 4). MEG3, which is downregulated in multiple cancer cell lines, is epigenetically activated upon the methylation of histone H3 at lysine 4 (H3K4me3) by MEN1 (multiple endocrine neoplasia type 1), a component of the MLL complex that functions as a tumor suppressor [78,193,269]. Thus, epigenetic changes can alter the expression of ncRNAs affecting downstream pathways, as well as other epigenetic factors, thereby establishing a complex network that is disrupted during carcinogenesis. This complex network could be altered in response to environmental changes and/or stress responses, including exposure to toxic pollutants (Figure 4) [4,5]. Ultimately, a better understanding of the mechanisms underlying the deregulation of ncRNAs will advance cancer treatments by serving as biomarkers for the early detection of cancers and for developing new clinical therapies.

Approximately 95% of the human genome represents non-coding sequences that are transcribed into ncRNAs capable of regulating gene expressions by multiple pathways, including post-transcriptional, translational and epigenetic mechanisms. Therefore, it is highly possible that ncRNAs play an essential role in maintaining the cell fate identity to prevent the transformation into malignancies. Hence, the deregulation of ncRNAs promote tumorigenesis by increasing cancer cell proliferation, CSC renewal, metastasis and therapy resistance. Rapidly advancing genome-wide technologies are facilitating the identification of novel ncRNAs and their regulatory mechanisms in the contexts of health and disease. Some of these mechanisms include the role of ncRNAs in modulating the three-dimensional (3D) genomic architecture [270]. Future studies are expected to elucidate the interlocking functions of ncRNAs with the epigenome and the 3D genomic architecture for developing new cancer therapies and/or earlier prognosis methods. 

## Figures and Tables

**Table 1:** MicroRNAs (MiRNAs) targeting epigenetic regulators. DNMT1: DNA-methyl transferase 1 and AML: acute myeloid leukemia.

*miRNA*	*Targets*	*Function*	*References*
*miR-29 a, b, c*	DNMT3A and DNMT3B	Tumor suppression by repression of de novo DNA methylation. Protects tumor-suppressor genes from been silenced by DNA methylation.	Fabbri et al., 2007 [44] Suzuki et al., 2013 [47]
*miR-148*	DNMT3B DNMT1	Negative feedback loop between DNMT1 and miR-148 in AML. Inhibition of cell proliferation and increase of apoptosis.	Duursma et al., 2008 [45] Wang et al., 2019 [48]
*miR-449a*	HDAC1	Inhibition of tumor growth, invasion and metastasis. Promotes apoptosis and differentiation.	Noonan et al., 2009 [49] Yong-Ming et al., 2017 [50]
*miR-152* *miR-185* *miR-342*	DNMT1	DNA hypomethylation. Promotes the expression of tumor-suppressor genes.	Suzuki et al., 2013 [47]
*miR-26a* *miR-98* *miR-124* *miR-214* *let-7* *miR-101* *miR-137*	EZH2	Prevents the progression of prostate cancer and metastasis.	Suzuki et al., 2013 [47]

**Table 2:** Long non-coding RNA (LncRNA) interactions with epigenetic regulatory complexes.

*lncRNA*	*Origin/Location*	*Interactions with Epigenetic Regulators*	*Function*	*References*
*HOTAIR* *(HOX transcript antisense RNA)*	Transcribed from antisense strand of homeobox C gene in chromosome 12	PRC2 LSD1/CoREST	Gene silencing by methylation of H3K27me3 and demethylation of H3K4me2	Cai et al., 2014 [65]
*SCHLAP1* *(second chromosome locus associated with prostate-1)*	From chromosome 2	SWI/SNF	Partially antagonizes location and function of SWI/SNF	Raab et al., 2019 [67]
*NEAT1* *(nuclear paraspeckle assembly transcript 1)*	Transcribed from the multiple endocrine neoplasia locus in chromosome 11	Subpopulation of SWI/SNF complexes	Nuclear paraspeckle (nuclear bodies) assembly	Neve et al., 2018 [69]
*XIST* *(X-inactive specific transcript)*	Chromosome X	PRC1	Silencing one pair of X chromosomes	Pintacuda et al., 2017 [63]
*ANRIL* *(antisense non-coding RNA in the INK4 locus)*	Transcribed from the CDKN2A/B gene cluster at chromosome 9 in the antisense direction	PRC1 (CBX7), PRC2 (SUZ12)	Transcriptional repression	Chi et al., 2017 [71]
*GAS5* *Growth arrest-specific 5)*	From chromosome 1	PRC2	Repression of glucocorticoids receptors, IRF4 (interferon regulatory factor 4)	Wang et al., 2018 [73]
*MEG3* *(maternally expressed 3)*	Maternally expressed, generates multiple isoforms by alternative splicing, from chromosome 14	JARID2, EZH2	Transcriptional repression	Wang et al., 2018 [73]
*PVT1* *(plasmacytoma variant translocation 1)*	From chromosome 8	PRC2 (EZH2)	Oncogene	Yu et al., 2018 [75]
*MALAT1* *(metastasis associated lung adenocarcinoma transcript 1)*	Also known as NEAT2 (non-coding nuclear-enriched abundant transcript 2). Infrequently spliced ncRNA, from chromosome 11	PRC2 (EZH2), HDAC9, BRG1	Tumorigenesis Vascular disease	Wang et al., 2018 [73] Cardenas et al., 2018 [77]
*KCNQ1OT1* *(KCNQ1 overlapping transcript 1)*	Part of an imprinting control region in chromosome 11	G9a, PRC2 (EZH2)	Gene silencing by H3K9me2 H3K27me3	Wang et al., 2018 [73]
*H19* *(H19 imprinted maternally expressed transcript)*	From imprinted region in chromosome 11	SAHH, PRC2 (EZH2)	Tumor-suppressor Oncogene	Zhou et al., 2015 [76]
*UCA1* *(urothelial cancer associated 1)*	From chromosome 19	PRC2 (EZH2), SWI/SNF	Tumorigenesis	Neve et al., 2018 [69]
*PANDAR* *(promoter of CDKN1A antisense DNA damage activated RNA)*	From chromosome 6	PRC1 PRC2	Tumorigenesis	Puvvula et al., 2014 [78]

**Table 3:** MiRNAs implicated in multiple cancers.

*miRNA*	*Cancer/Disease Involvement*	*References*
*miR-15b* *miR-16*	Upregulated in gastric cancer and downregulated in chronic lymphocytic leukemia (CLL)	Xia et al., 2008 [79] Cimmino et al., 2005 [80] Xia et al., 2008 [79]
*LET-7*	Downregulated in lung, pancreatic cancer and acute lymphoblastic leukemia (ALL)	Takamizawa et al., 2004 [81] Kugel et al., 2016 [82]
*miR-34 (a, b and c)*	Downregulated in gastric and cervical cancer neuroblastoma. Upregulated in glioblastoma multiforme (GBM) (*miR-34b*) and colorectal cancer (miR-34a)	Zhang and Liao, 2019 [83] He et al., 2009 [84], Hermeking et al., 2012 [85] Bommer et al., 2007 [86] Tarasov et al., 2007 [87] He et al., 2009 [84] Hasakova et al., 2019 [88] Han et al., 2002 [89]
*miR-21*	Upregulated in GBM, solid tumors and multiple myeloma	Kumarswamy et al., 2011 [90] Asangani et al., 2008 [91] Wang et al., 2019 [92] Jesionek-Kupnicka et al., 2019 [93] Pfeffer et al., 2015 [94]
*miR-125 (a and b)*	Upregulated in AML and GBM (miR-125b)	Bousquet et al., 2010 [95] Chaudhuri et al., 2012 [96] Wu et al., 2013 [97] Romero et al., 2015 [98] Liu et al., 2017 [99] Jesionek-Kupnicka et al., 2019 [93]
*miR-181d*	Downregulated in GBM	Zhang et al., 2012 [100] Yang et al., 2018 [101] Jesionek-Kupnicka et al., 2019 [93]
*miR-648*	Downregulated in GBM	Kreth et al., 2013 [102] Jesionek-Kupnicka et al., 2019 [93]
*miR-155*	Upregulated in AML, colorectal cancer and Hodgkin’s lymphoma	Fabbri et al., 2008 [103] Narayan et al., 2017 [104] Witten and Slack, 2020 [105] Kluiver et al., 2005 [106] Narayan et al., 2018 [107] Eis et al., 2005 [108]
*miR-221*	Upregulated in GBM	Lukiw et al., 2009 [109]
*miR-30a-5p*	Downregulated in colorectal cancer	Wei et al., 2016 [110]
*miR-29 family*	Upregulated in colorectal and cervical cancer and downregulated in lung cancer and AML	Fabbri et al., 2007 [44] Jiang et al., 2014 [111]
*miR-145*	Downregulated in colorectal cancer	Michael et al., 2003 [112] Sheng et al., 2017 [113]
*miR-128a*	Upregulated in AML	De Luca et al., 2017 [114]
*miR-17/92 cluster*	Upregulated and downregulated in myeloid leukemias and upregulated in colorectal cancer and CLL	Fabbri et al., 2008 [103] Jiang et al., 2011 [115] Moussay et al., 2011 [116] Willimott and Wagner, 2012 [117] He et al., 2013 [118]
*miR-7*	Downregulated in GBM	Luo et al., 2015 [119]
*miR-185*	Downregulated in GBM	Zhang et al., 2011 [120]
*miR-24a*	Upregulated in AML	Fabbri et al., 2008 [103]
*miR-200*	Downregulated in breast cancer	Mekala et al., 2018 [121]
*miR-150*	Downregulated in CTCL, AML	Jiang et al., 2012 [122] Ito et al., 2014 [123] Abe et al., 2017 [124]

**Table 4:** LncRNAs implicated in diverse cancers. T-ALL: T-cell acute lymphoblastic leukemia.

*lncRNA*	*Cancer Involvement*	*References*
*PVT1*	Gastrointestinal, renal, breast cancer, acute promyelocytic leukemia	Martínez-Barriocanal et al., 2020 [171] Wang et al., 2019 [172] Sun et al., 2015 [173] Zeng et al., 2015 [174]
*HOXD1-AS1*	Bladder, cervical, gastric, ovarian, colorectal, prostate, GBM, melanoma, osteosarcoma, liver and non-small-cell lung cancers	Braga et al., 2020 [175] Wang et al., 2017 [176] Chi et al., 2018 [177] Yang et al., 2019 [178]
*HOTAIR*	Pancreatic, cervical, breast, lung, oral, gastric cancers, AML	Liu et al., 2013 [179] Liu et al., 2014 [180] Xing et al., 2015 [181] Bhan et al., 2015 [182] Zhang et al., 2018 [183] Hajjari and Salavaty, 2015 [184]
*SPRY4-IT1*	Breast and cervical cancer	Li et al., 2017 [185] Shi et al., 2015 [186]
*GAS5*	Breast, lung, prostate cancer, blood	Pei et al., 2015 [187] Xu et al., 2016 [188] Ji et al., 2019 [189]
*PANDAR*	Breast, gastric, colorectal, bladder cancer	Sang et al., 2016 [190] Zou et al., 2018 [191]
*MEG3*	Gastric and pancreatic cancer, AML	Benetatos et al., 2010 [192] Modali et al., 2015 [193] Jiao et al., 2019 [194] Bhan et al., 2017 [170]
*SNHG1*	Cervical cancer	Liu et al., 2018 [195]
*CCAT-1*	Colon, gastric cancer, AML	Li et al., 2019 [196]
*H19*	Breast, gastric cancer	Wang et al., 2020 [197] Ghafouri-Fard et al., 2020 [198]
*UCA1*	Pancreatic, colorectal cancer, AML	Neve et al., 2018 [69] Hughes et al., 2015 [199]
*MALAT1*	Lung, cervical, breast cancer, lymphoblastic leukemia	Sun et al., 2016 [200] Yang et al., 2015 [201] Tripathi et al., 2010 [202]
*SChLAP1*	Prostate cancer	Prensner et al., 2013 [66]
*NEAT1*	Breast, gastric, colorectal cancer, acute promyelocytic leukemia	Zeng et al., 2014 [203] Zhang et al., 2019 [204]
*ANRIL*	Gastric cancer, breast cancer, adult T-cell leukemia	Meseure et al., 2016 [72] Liu et al., 2018 [205] Song et al., 2018 [206]
*LUNAR1*	T-ALL, lymphoblastic leukemia	Trimarchi et al., 2014 [207]
*IRAIN*	AML	Sun et al., 2014 [208]
